# In-Situ Studies of Structure Transformation and Al Coordination of KAl(MoO_4_)_2_ during Heating by High Temperature Raman and ^27^Al NMR Spectroscopies

**DOI:** 10.3390/ma10030310

**Published:** 2017-03-17

**Authors:** Min Wang, Jinglin You, Alexander Sobol, Liming Lu, Jian Wang, Yingfang Xie

**Affiliations:** 1 State Key Laboratory of Advanced Special Steel & Shanghai Key Laboratory of Advanced Ferrometallurgy & School of Materials Science and Engineering, Shanghai University, Shanghai 200072, China; wangmin1558@126.com (M.W.); wj581692@126.com (J.W.); yingfangxie@163.com (Y.X.); 2 Prokhorov General Physics Institute, Russian Academy of Sciences, Ulitsa, Vavilova 38, Moscow 119991, Russia; 3 CSIRO Mineral Resources, Technology Court, Pullenvale, Queensland 4069, Australia

**Keywords:** double molybdate KAl(MoO_4_)_2_, structure transformation, Al coordination environment, in-situ Raman spectroscopy, first principles calculation, ^27^Al MAS NMR

## Abstract

Recent interest in optimizing composition and synthesis conditions of functional crystals, and the further exploration of new possible candidates for tunable solid-state lasers, has led to significant research on compounds in this family M^I^M^III^(M^VI^O_4_)_2_ (M^I^ = alkali metal, M^III^ = Al, In, Sc, Fe, Bi, lanthanide; M^VI^ = Mo, W). The vibrational modes, structure transformation, and Al coordination of crystalline, glassy, and molten states of KAl(MoO_4_)_2_ have been investigated by in-situ high temperature Raman scattering and ^27^Al magic angle spinning nuclear magnetic resonance (MAS NMR) spectroscopy, together with first principles density functional simulation of room temperature Raman spectrum. The results showed that, under the present fast quenching conditions, Al is present predominantly in [AlO_6_] octahedra in both KAl(MoO_4_)_2_ glass and melt, with the tetrahedrally coordinated Al being minor at approximately 2.7%. The effect of K^+^, from ordered arrangement in the crystal to random distribution in the melt, on the local chemical environment of Al, was also revealed. The distribution and quantitative analysis of different Al coordination subspecies are final discussed and found to be dependent on the thermal history of the glass samples.

## 1. Introduction

Molybdates and tungstates doped with transition metal or rare earth ions are important compounds that are found to have potential in tunable laser applications [1,2,3]. Double molybdates, with their general formula of M^I^M^III^(M^VI^O_4_)_2_ (M^I^ = alkali metal, M^III^ = Al, In, Sc, Fe, Bi, lanthanide; M^VI^ = Mo, W), have been studied extensively due to their unique optical and antiferroelectric properties and ferroelastic phase transitions [4,5,6]. Some of its members behave as promising laser host materials for transition metal and lanthanide ions due to their high and continuous transparency in the wide range of the near-IR region. For instance, Cr^3+^-doped KAl(MoO_4_)_2_ crystal has been proven to be a suitable laser host, characterized by high absorption, efficient pumping, and broad laser emission [7]. As a member of M^I^M^III^(M^VI^O_4_)_2_, KAl(MoO_4_)_2_ represents the large crystal family with layered structure. It has attracted considerable interest since many compounds of this series exhibit a sequence of ferroelastic phase transitions and are therefore of great importance for basic research [8,9].

Information on melt structure, including coordination species of highly charged cations and the basic structure units, will assist in optimizing the composition and synthesis conditions of functional crystals, and thereby looking for new possible candidates as tunable solid-state lasers [10]. Hence, more complete data on melt structure and properties will benefit possible future applications of M^I^M^III^(M^VI^O_4_)_2_ compounds with layered structure. The basic structure units and the form of cluster structures present in molten alkali metal molybdates/tungstates K_2_M*_n_*O_3*n*+1_ (*n* = 1, 2, 3, 4) have been determined, in which Mo and W were found to be present only in tetrahedral coordination [11,12,13,14]. Although Voron’ko et al. [15,16] pointed out earlier that the [WO_4_]^2−^ group is more likely to be coordinated to rare earth ions than to alkali metal ions in mixed molten tungstates, the coordination chemistry and structural role of trivalent cations in molten M^I^M^III^(M^VI^O_4_)_2_ double molybdates/tungstates remain unclear, let alone the more complex situation of rare earth ions. To date, no such results have been obtained on the coordination species of trivalent cations in the family of M^I^M^III^(M^VI^O_4_)_2_ in glassy and molten states. Hence, aluminum-bearing double molybdate was first chosen for the study, with the purpose of acquiring information on the local coordination environment and distribution of Al, as well as the structural significance of alkali metal ions present in the molten double molybdate.

Vibrational spectroscopy (IR and Raman), has been used in previous studies to determine the coordination species of Si and Al in the glass and melt of aluminosilicates [17,18,19]. However, the vibrational properties of glass and melt are not understood well enough to unambiguously assign the features in the spectra to specific coordination polyhedra. The MAS NMR spectroscopy has provided an excellent technique for investigating the structural properties of glass because its chemical shifts are extremely sensitive to the local environment of the nucleus, especially the nearest neighbour coordination number and geometry. ^27^Al MAS NMR can therefore provide the environmental information of Al ions and offer important insight into the Al species and their sub-species in amorphous glasses [20]. On the basis of previous studies, the chemical shift values of different coordinated Al species in various inorganic salt systems determined by ^27^Al NMR were summarized in Table 1. However, only a single, averaged resonance was observed on the timescale of the NMR experiment (10^−9^–10^−7^ s^−1^) in molten aluminosilicates, likely due to rapid chemical exchange occurring between different silicate and aluminate species [21]. Similarly, when investigating molten Al_2_O_3_ at 2323 K, Coutures et al. [22] observed rapid chemical exchanges (on the order of about 10^−9^ s^−1^) between the aluminate species in the melt. As a result, only a single narrow resonance was observed. In summary, NMR spectroscopy reveals useful but limited information on the nature of structural species present in high temperature melts.

While glass structure can be studied conveniently and precisely at ambient temperature, it is much more challenging to investigate the liquid structure at high temperature due to the extreme conditions applied (high temperature, high volatility) [23,24]. Glasses are often described as a frozen image of their corresponding high temperature liquids at a certain “fictive temperature” (*T*_f_). The fictive temperature of a substance is defined where the structure of melt and glass is essentially equal. The final glass structure is partially dictated by its fictive temperature, depends on the cooling rate *q*_c_ as evidenced in $T_{f}\;\propto \;\ln\left\{q_{c}\right\}$ [25,26,27]. Therefore, KAl(MoO_4_)_2_ glasses were used as the supplementary to study the structure of molten KAl(MoO_4_)_2_.

In the present paper, the structure and local Al coordination environment of crystalline, amorphous and molten KAl(MoO_4_)_2_, whose composition belongs to the K_2_O-Al_2_O_3_-MoO_3_ ternary system, were studied using in-situ high temperature Raman and NMR spectroscopies.

## 2. Materials and Methods

### 2.1. Material Preparation

The polycrystalline compound KAl(MoO_4_)_2_ was prepared by conventional solid-state reaction [55,56,57,58] from analytical grade reagents K_2_CO_3_, Al_2_O_3_, and MoO_3_ (all from Sinopharm Chemical Reagent Co., Ltd., Shanghai, China), which were mixed at a stoichiometric ratio and calcined at 873 K for 40 h in a platinum crucible, and then slowly cooled to room temperature at a cooling rate of 0.24 K/min (Supplementary Materials Table S1).

Since the melt is highly viscose, it is not easy to prepare the pure amorphous state KAl(MoO_4_)_2_ by the traditional quenching technique. After the mixture of K_2_CO_3_, Al_2_O_3_, and MoO_3_ were melted in a muffle furnace at 1173 K (Table S2), the “hammer-and-anvil” technique (by liquid quenching between two metallic plates) described by Voronko et al. [12] was therefore used in the present study to ensure the cooling rate required to synthesize the KAl(MoO_4_)_2_ glass. In this work, two kinds of the as-quenched glass were prepared under fast and slow quenching conditions.

### 2.2. Material Characterization

The structure of crystalline and amorphous states of the samples synthesized were characterized by powder X-ray diffraction, which was performed on a D8 ADVANCE powder diffractometer (Bruker AXS, Karlsruhe, Germany), using Cu K*α* radiation (*λ* = 1.5418 Å) with a step size of 0.02°.

Temperature-dependent studies of Raman spectra were performed on a Horiba Jobin Y’von LabRAM HR800 micro_Raman spectrometer equipped with a microscopic heating furnace (Linkam, TS1500, Epsom, UK) with a temperature deviation about ±1 K. A 355 nm ultraviolet pulse laser beam operating at 100 mW was used as an excitation source. The slits were set to achieve a resolution of 2 cm^−1^.

The ^27^Al MAS NMR spectra were obtained using a Bruker AVANCE III 400 WB spectrometer operating at 9.4 T with a spinning rate of 10 kHz. A recycle delay of 2 s was used along with a 4 mm double resonance MAS probe. The chemical shift values for ^27^Al nuclei were determined with reference to a 1 M aqueous solution of Al(NO_3_)_3_.

### 2.3. Computational Simulation

The room temperature Raman spectrum of KAl(MoO_4_)_2_ crystal was simulated using the Cambridge Sequential Total Energy Package (CASTEP, Cambridge, UK), which employed the plane wave pseudopotential formalism of density functional theory (DFT) and was specifically designed to investigate crystal properties [59,60,61]. The electron correlation effects were modeled using the Wu-Cohen (WC) [62] generalized gradient approximation (GGA). A plane wave cut-off energy of 830.0 eV and the default “ultrasoft” [63] norm-conserving pseudopotentials of CASTEP 5.5 were applied for the geometry optimization. The convergence thresholds were set: at an energy tolerance of 5.0 × 10^−6^ eV/atom, a maximal force tolerance of 0.01 eV/Å, a maximal displacement of 5.0 × 10^−4^ Å, and a maximal stress of 0.02 GPa.

## 3. Results and Discussion

### 3.1. Crystallinity and Molecular Structure of Crystalline KAl(MoO_4_)_2_

**Figure 1:**
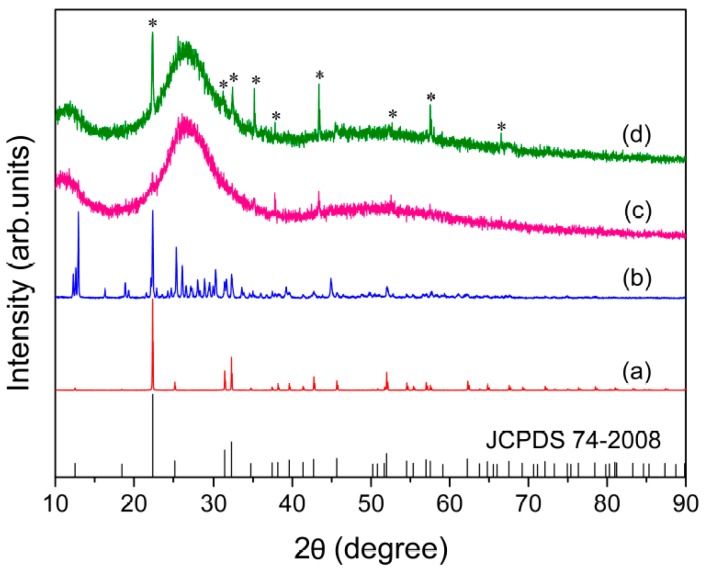
X-ray diffraction patterns of the powdered bulk samples: (**a**) Synthesized at 873 K for 40 h; (**b**) fast quenched and then annealed at 723 K for 80 min; (**c**) fast; and (**d**) slow quenched after being melted at 1173 K. The asterisks indicate the trace peaks due to the crystalline phase.

**Figure 2:**
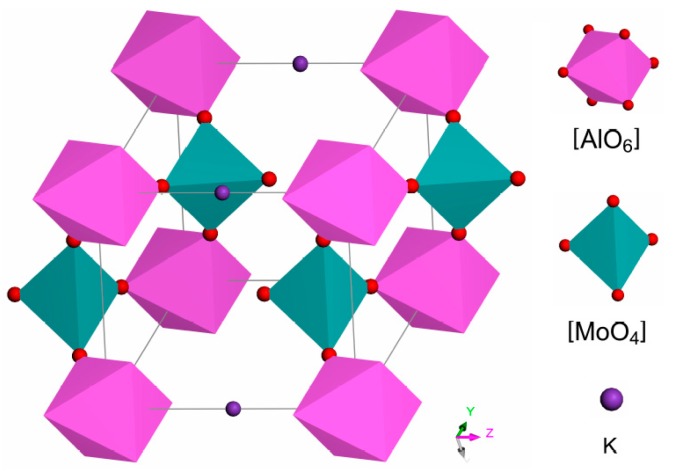
Unit cell of KAl(MoO_4_)_2_ crystal in the hexagonal phase [P$\bar{3}$m1 (*D*_3*d*_^3^)] space group.

The powder X-ray diffraction was used to confirm the crystallinity of the KAl(MoO_4_)_2_ sample synthesized under slow cooling conditions. As demonstrated in Figure 1a, the sample prepared through slow cooling was characterized by excellent crystallinity with its pattern in good agreement with a hexagonal phase (74-2008, JCPDS). The crystalline structure of KAl(MoO_4_)_2_ is described by the P$\bar{3}$m1 (*D*_3*d*_^3^) space group, with one formula per unit cell and lattice parameters of *a* = 5.545, *c* = 7.070 Å [2]. In the structure, each Mo ion is surrounded by four oxygen ions with a tetrahedral coordination, while the Al^3+^ surrounded with six oxygen ions in an octahedral coordination. The [AlO_6_] octahedra share corner oxygen ions with adjacent [MoO_4_] tetrahedra, forming a layered structure with K^+^ orderly situated between these layers for charge compensation, as represented in Figure 2.

**Figure 3:**
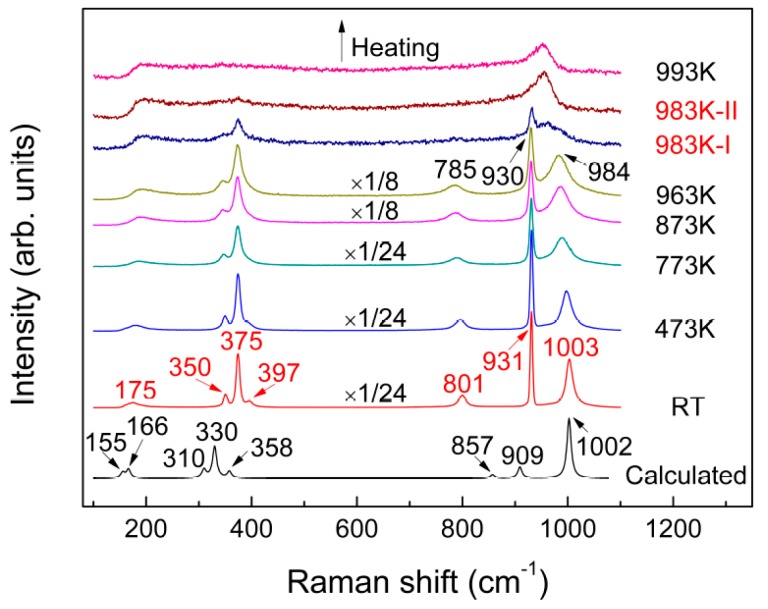
Temperature-dependent Raman spectra of KAl(MoO_4_)_2_ recorded from RT to 993 K and the room temperature spectrum calculated by the Cambridge Sequential Total Energy Package (CASTEP).

Figure 3 shows the room temperature Raman spectrum of the same sample. Seven Raman bands were observed that can be probably assigned to the internal stretching modes (above 800 cm^−1^) and external lattice vibrations (less than 400 cm^−1^). The room temperature Raman spectrum was also calculated using CASTEP and compared with the experimental spectrum in Figure 3, with the atomic coordinates of KAl(MoO_4_)_2_ used for calculating Raman vibrational modes listed in Table 2. The calculated results correlate well with the experimental data, both in the position and intensity of the characteristics bands, after the wavenumbers were corrected with a scaling factor of 0.9441. Based on the calculated results, the corresponding assignment of various vibrational modes is listed in Table 3.

**Figure 4:**
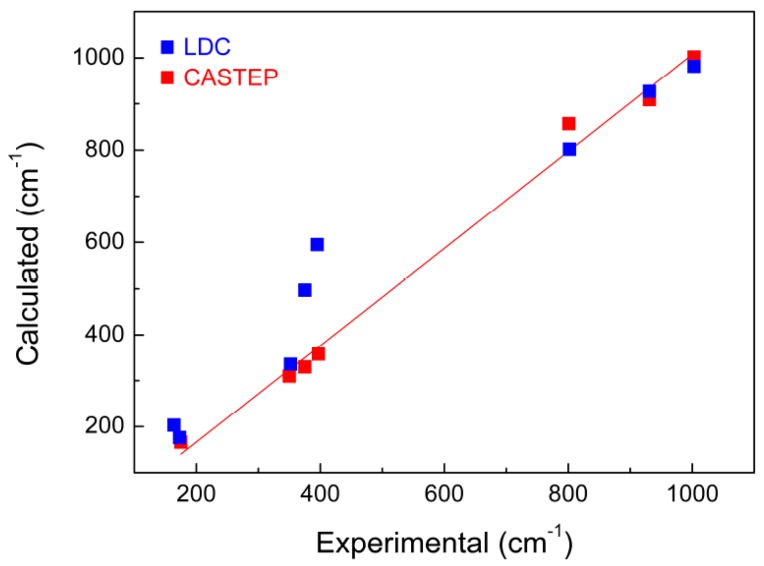
Experimental and calculated wavenumbers of KAl(MoO_4_)_2_ crystal by CASTEP (red squares) in this work and LDC (blue squares) carried out by Maczka et al. [8]

Compared with the results predicted by lattice dynamics calculations (LDC) (blue squares in Figure 4, which were carried out by Maczka et al. [8]), the wavenumbers of KAl(MoO_4_)_2_ calculated by CASTEP in this work on the basis of DFT is clearly more satisfied. However it is not clear whether this conclusion can be generalized to other molybdate systems. Major differences in intensity between the experimental and calculated results were observed at the peak positions of 909 and 1002 cm^−1^, which is believed to be due to the precision and limitation of the theoretical simulation methods.

### 3.2. Structural Transformation of KAl(MoO_4_)_2_ Samples During Heating and Melting

#### 3.2.1. Structure Evolution of Crystalline KAl(MoO_4_)_2_ with Temperature

The effect of temperature on the shape and position of characteristics bands was observed from RT to 963 K in Figure 3. As the sample temperature increased, nearly all peaks were found to be gradually diffused and showed red-shift, which originated from broadened distributions of the bond angles and increased bond distances between the atoms. Further increase in the sample temperature to 983 K witnessed remarkable changes in Raman spectrum, suggesting occurrence of the melting process of the sample. As can be seen from Figure 3, the band at 785 cm^−1^ disappeared during the melting process, while the band at 984 cm^−1^ merged with a neighbouring peak at 930 cm^−1^, to form a new, more diffused vibrational band approximately at about 953 cm^−1^. Taking into account the bands at 931 and 1003 cm^−1^ at room temperature that represent stretching vibrations of Mo–O and Al–O–Mo, respectively, the observed changes demonstrated that the bonds of Mo–O and Al–O–Mo tend to be consistent and homogenized. As will be discussed systematically and in detail later, the significant changes of Raman spectra observed suggested considerable variations in K^+^ arrangement, chemical environment of Al and Mo, as well as distortion of [AlO_6_] and [MoO_4_], during melting.

**Figure 5:**
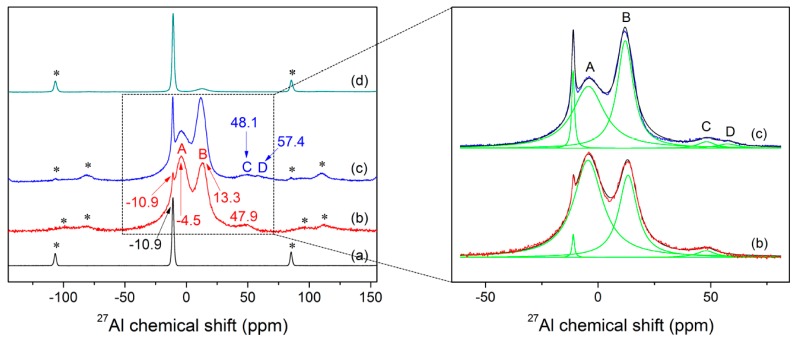
^27^Al MAS NMR spectra recorded at room temperature for the KAl(MoO_4_)_2_ samples prepared under different conditions: (**a**) slow cooling; (**b**) quenched at fast cooling; (**c**) quenched at a slow cooling rate; (**d**) quenched at fast cooling rate and then annealed at 723 K for 80 min, as well as the deconvolution of NMR spectra of fast and slow quenched samples (on the right part). The bands labelled with asterisks are related to spinning sidebands.

The room temperature ^27^Al MAS NMR spectrum of KAl(MoO_4_)_2_ sample obtained under slow cooling condition is shown in Figure 5a. The spectrum shows a peak at −10.9 ppm which corresponds to the six-coordinated Al. As is generally known, the symmetry differences will lead to changes in chemical shift and linewidth [64]. For example, the MAS NMR spectroscopy revealed only two inequivalent Al signals (−11.1 and −13.8 ppm) for crystalline Al_2_(MoO_4_)_3_ [65], while it had four magnetically inequivalent Al types (−11.38, −11.67, −13.50 and −14.06 ppm) as proved by double-rotation NMR [66]. Although Al is present in six coordination at the room temperature for both KAl(MoO_4_)_2_ and Al_2_(MoO_4_)_3_, the ^27^Al MAS NMR spectrum of KAl(MoO_4_)_2_ shows only a single resonance, demonstrating the ordered structural arrangement of Al at the equivalent site.

#### 3.2.2. Structure of As-quenched KAl(MoO_4_)_2_ and Its Evolution with Temperature

As can be seen from Figure 1c,d, the as-quenched samples obtained under fast and slow quenching conditions showed typical XRD spectra consisting of extremely diffused bands characterized by amorphous materials, suggesting the complete vitrification was almost achieved under the conditions used in the present study. The samples contained only a trace quantity of crystalline material, as evidenced by minor peaks at the peak positions of crystalline KAl(MoO_4_)_2_ (see Figure 1a). However, the presence of a minor amount of crystalline material is not expected to have any significant effect on the subsequent experimental analysis and the results.

**Figure 6:**
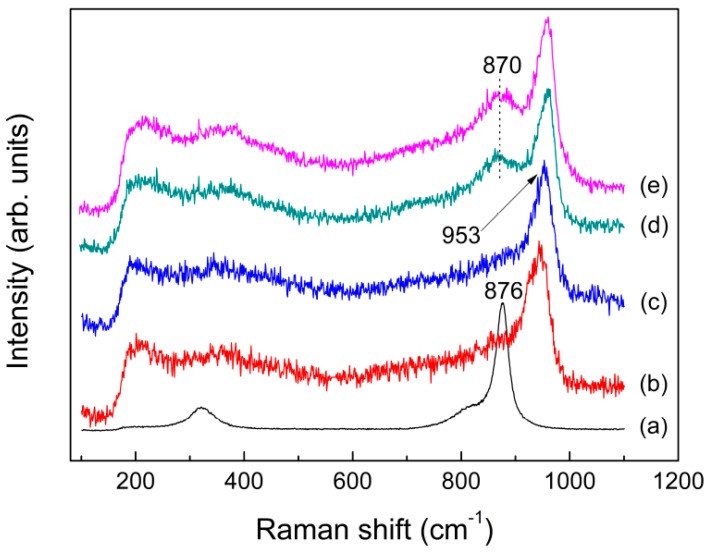
Raman spectra of molten: (**a**) K_2_MoO_4_ at 1273 K; (**b**) K_2_Mo_4_O_13_ at 1023 K; (**c**) KAl(MoO_4_)_2_ at 993 K; (**d**) fast; and (**e**) slow quenched KAl(MoO_4_)_2_.

As previously described in the Introduction section, the faster melt-quench rates, the higher *T*_f_ values and the more disordered glass structure. The structural relationship between glass and melt was investigated on a variety of aluminosilicates by high temperature Raman spectroscopy, which has revealed the same anionic species both in the quenched glass and melt and thereby confirmed previous conclusions about the structure of melt made on the basis of measurements on the quenched glass [67]. The key differences between the quenched glass and melt are shown in the position and intensity of their Raman lines. It was observed from Figure 6c–e that, compared with the as-quenched glass, the major vibrational bands of KAl(MoO_4_)_2_ melt at about 953, 870 and 700 cm^−1^, are slightly shifted toward lower frequencies, resulting from the increasing average bond lengths of Al–O–Mo (Al–O, Mo–O) and Mo=O with increasing temperature. In addition, a remarkable difference in Raman intensities between the quenched glass and melt was observed for the band at around 870 cm^−1^, which is probably caused by the asymmetric stretching vibrations of Al–O–Mo. As discussed later, this band corresponds to the band B in ^27^Al MAS NMR spectra (see Figure 5). The observed discrepancies between the quenched glass and melt suggested that the “frozen-in” anisotropy fluctuations might be of much greater in the quenched glass than what occurred in the melt. The excess Raman intensity of the band at around 870 cm^−1^ observed might be associated with a structural defect in the glass. As explained by Denisov et al. [68] for inorganic glass, like nitrate (Ca,K)(NO_3_)_3_ and borate (Li_2_O)_0.3_(B_2_O_3_)_0.7_, the stressed bonds arose in the disordered system of bonds which formed in the glass during quenching of the melt. That is, when a melt was rapidly cooled, the thermal motion of atoms was frozen, and thus a region of localized deformation internal stress arose which generated from a “switched-bond defect”. In the case of KAl(MoO_4_)_2_ glass, the asymmetry stretching vibrations of Al–O–Mo compared to the symmetry stretching vibrations of Mo=O are particularly sensitive to the structural defect.

Although the chemical ratio of KAl(MoO_4_)_2_ is same with K_2_MoO_4_, the band shape and position of its melt spectrum were quite similar to those for K_2_Mo_4_O_13_, as shown in Figure 6a–c. This demonstrate that the melt structure of KAl(MoO_4_)_2_ due to the presence of Al has been rather different from the isolated [MoO_4_] tetrahedra existed in molten K_2_MoO_4_, which in turn indicate the role of Al is not present as the simple metal cations like potassium ions. In molten aluminosilicates, the Raman frequency of non-bridging oxygen Si–O increases due to the presence of six-coordinated Al, while shifting to lower wavenumbers in the presence of the tetrahedrally coordinated Al [69,70]. It is also observed for molten K_2_MoO_4_, with the band at 876 cm^−1^ shifted rightwards to 953 cm^−1^ in the melt spectrum of KAl(MoO_4_)_2_. As a result, Al probably exist as six coordination in the melt of KAl(MoO_4_)_2_. Detailed explanation on the mechanism of this phenomenon will be given in another article.

Figure 5b,c show the ^27^Al NMR spectra of as-quenched samples which contained little crystals, 1.1% and 5.1% for fast and slow quenched glass, respectively. As the quenching rate of sample (b) is a little faster than that of sample (c), sample (c) can be interpreted to be transformed from (b) over a certain period of time. Note that the quenching rate has a large effect on the NMR spectra of the glass obtained. The spectra (b, c) show two strong signals approximately at −4.5 and 13.3 ppm, and broad bands at about 48 and 57 (only observed in spectrum c) ppm, besides the peaks contributed by crystals. Based on the chemical shift values and their relative differences (Δ*δ*_i_) between Al^VI^ and Al^V^, Al^V^, and Al^IV^ summarized especially for various inorganic glass systems in Table 1 by ^27^Al MAS NMR, the observed peaks at −4.5 and 13.3 ppm, 48 and 57 ppm, can be attributed to the six- and four-coordinated Al, respectively. It is worth noting that the peak positions of Al in aluminophosphates in Table 1 were shifted upfield (more shielded) relative to the chemical shifts reported for aluminoborates and aluminosilicates glass, ranging from −25~−10, 6~15, and 22~45 ppm for hexa-, penta-, and tetra-coordinated Al species, respectively, either in crystalline or amorphous aluminophosphate materials. This is because the ^27^Al chemical shift was principally influenced by the electronegativity effect of the ligand, in this case due to the greater electronegativity, smaller atomic radius and larger field strength of P^5+^ compared to B^3+^, Si^4+^, and Mo^6+^, and reflected the change of the electronic densities [49,50]. The spectrum (b) has a featureless and asymmetric band centered at about 47.9 ppm, while shows two broad bands labeled as C and D in Figure 5c which corresponding to 48.1 and 57.4 ppm, respectively. According to the deconvoluted results of NMR spectra (b, c) (see Figure 5 on the right part), the band A and B accounted for about 63.4% and 33.9% in spectrum (b), and about 50.7% and 44.1% of the total amorphous peak area for spectrum (c), respectively. The broad band at around 47.9 ppm accounted for about 2.7%, while the band C and D contributed about 2.9% and 2.3% to the total amorphous peak area, respectively. In fact, the amount of [AlO_4_] species might be higher in the melt than that observed in the glass. However, the Raman spectra in Figure 6c–e do not obviously show the presence of tetrahedral aluminum, which is probably due to its low content.

**Figure 7:**
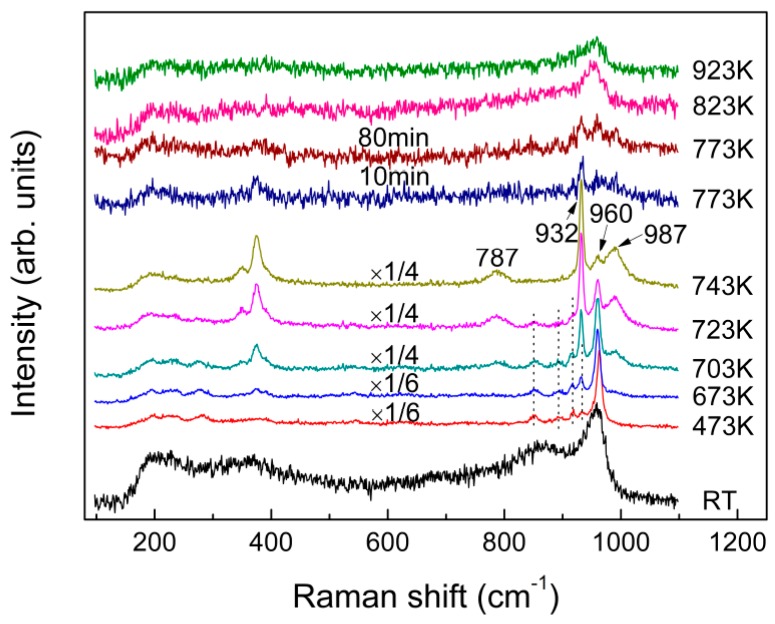
In-situ Raman spectra of the as-quenched (fast quenched) KAl(MoO_4_)_2_ sample recorded from RT to 923 K.

Figure 7 compares the in-situ Raman spectra of the fast-quenched sample recorded at various temperatures from room temperature to 923 K. As shown in Figure 7, new peaks started to appear at 851, 894, 918, 933, and 962 cm^−1^, as the quenched glass was heated to 473 K. With further increase in sample temperature to 743 K from 473 K, the band at 960 cm^−1^ gradually split into two peaks at 932 and 987 cm^−1^. As a result, the band at 932 and 987 cm^−1^ were enhanced with temperature before occurrence of the melting process at 773 K. Interestingly, the line shapes and varying trend of the three peaks at 932, 960 and 987 cm^−1^ with increasing temperature from 473 K to 743 K are similar with what occurred for the effect of quenching rate and annealing on the three main peaks at −10.9, −4.5 and 13.3 ppm of the NMR spectra in Figure 5b–d, while the Raman peak at 960 cm^−1^ finally became dominated after the sample being melted. In addition, another peak at 787 cm^−1^ started to appear at 703 K and it grew slightly as the sample temperature increased. It is well known that the glass will crystallize when being annealed, followed by structural ordering, and an increase in atomic motions and interatomic distances. Consequently, the atoms are capable to shift larger displacements and the internal stress near “switched-bond defect” begins to relax. Finally, the sample was converted to a state with a minimal deformation stress from the structural network of glass. Furthermore, the fast-quenched sample after being annealed at 723 K for 80 min is examined by XRD, as shown in Figure 1b, which cannot be simply indexed to any identified crystal phases. Therefore, the Raman peaks at 851, 894, 918, and 962 cm^−1^ observed at 723 K are believed to be related to some metastable phase, even though the attribution of their corresponding vibrational modes remains unknown. Figure 5d shows the ^27^Al NMR spectrum of the fast-quenched sample after being annealed at 723 K for 80 min, which has an asymmetric and featureless band at around 13 ppm together with the peak related to crystallization. It can be determined that this metastable phase is a transition state intermediate between glassy and crystalline states. Compared the peaks in Figure 5a,d, the band at around 13 ppm could be qualitatively confirmed as the six-coordinated Al in metastable phase. In addition, according to their temperature-dependent Raman spectra, the melting point of the fast-quenched sample was about 160 K lower than that of the crystalline sample. Since the fast-quenched sample was prepared under rapid cooling conditions, it contained considerable residual defects and stresses. Thus, the sample was thermodynamically unstable and is easier to melt compared with the crystalline sample prepared through slow cooling.

### 3.3. Chemical Environment and Coordination of Al in KAl(MoO_4_)_2_ Samples Prepared Under Different Conditions

From Figure 5b–d, the band A disappeared progressively and the crystallization peak at −10.9 ppm enhanced gradually, whereas the band B became slightly stronger before being depressed. The as-quenched sample has shown a clear trend that band A gradually transformed into the crystallization peak, suggesting the evidence of crystallization. The intensity of band B reduced sharply with only a small diffused band left after the fast-quenched sample was annealed at 723 K for 80 min, while band A disappeared completely. It should be mentioned clearly that the band B was not observed in the slowly cooled sample (Figure 5a) and almost disappeared after annealing (Figure 5d). In contrast, band A turned to the sharp peak at −10.9 ppm both when the sample was cooled slowly and annealed. Considering the samples prepared under different thermal histories, the chemical coordination of Al in glassy and molten states of KAl(MoO_4_)_2_ could be confirmed definitely. That is, band A is attributed to the molten state Al^VI^ species, while the intermediate state Al^VI^ species is mainly represented by band B. What’s more, it was found that quenching rate had a considerable impact on the occurrence and distribution of different Al subspecies present in the quenched samples. Therefore it is important to ensure sufficient quenching rate to prepare the complete vitrification materials.

It is generally believed that for some cation nuclei such as ^29^Si and ^27^Al, their *δ*_i_ decreases with increasing coordination number and accordingly increasing mean bond distance of cation-oxygen, which reflects a lower electron density around the cation with increasing coordination number [71]. For the smaller, more highly charged cations, like Si and Al, their chemical shifts are generally influenced through modification of the electron distributions around the intervening oxygens by the first neighbour cations [72]. However, there has been relatively less attention directed towards the structural significance of alkali metal ions compared with the network formers, such as Mo and Si. The geometry of the K^+^ in KAl(MoO_4_)_2_ crystal is to a large extent determined by [MoO_4_] and [AlO_6_], whereas more freedom of K^+^ is expected in the melt. In the crystalline state, the potassium ions are regularly distributed between layers of [MoO_4_] tetrahedra which are further connected with [AlO_6_] octahedra. The nearest neighbours to potassium ions are the six intervening oxygens of Al–O–Mo (K–O = 2.850 Å), closer than the six non-bridging oxygens of Mo–O (K–O = 3.215 Å). The distance between Al and its first neighbouring K^+^ cations in the crystal is 3.535 Å, compared with the nearest atomic distance of 3.606 Å between Al and Mo. Once the crystal starts to melt, K^+^ ions, being network modifiers, first deviate from their equilibrium positions and then move more freely to be distributed homogeneously in the melt. In other words, the distribution of K^+^ is no longer limited to the interlayers, but they are present as free ions. The octahedral Al species in molten state probably have equivalent Mo and K ions as the first neighbouring cations. When the melt was removed from the muffle furnace and quenched in air by the “hammer-and-anvil” technique, there was not enough time for the randomly distributed K^+^ ions to be transformed into their regular positions between layers in the crystal. The nearest neighbours to potassium ions might probably be the non-bridging oxygens of Mo–O and Mo=O in the metastable phase. The chemical environment of Mo is influenced by the first neighbouring potassium cations, and then affected the chemical shifts of Al^VI^ through modification of the electron distribution around the intervening oxygen atoms of Al–O–Mo. Thus, the Raman band at about 870 cm^−1^ of the as-quenched samples in Figure 6 has also been confirmed, which was actually due to the chemical environment variation around Al–O–Mo.

From in-situ high temperature Raman and ^27^Al MAS NMR spectroscopic studies, it was confirmed that Al is predominantly present in octahedral species in the KAl(MoO_4_)_2_ glass and melt, with a small amount in tetrahedral coordination. Note that although the octahedral Al species are the same for the crystalline, metastable, and molten states of KAl(MoO_4_)_2_, the local chemical environment and distribution of the corresponding subspecies can vary considerably due to the distortion of the framework which originated from the distribution of K^+^ in the alkali sites. The molten KAl(MoO_4_)_2_ can be proposed to be a typical glass-forming liquid due to the highly cross-linked network of [MoO_4_] tetrahedra and [AlO_6_] octahedra.

## 4. Conclusions

The vibrational modes are studied through a first principles density functional simulation of the Raman spectrum for the crystal. The room temperature Raman spectrum of KAl(MoO_4_)_2_ crystal calculated by CASTEP correlates well with the experimental results. In-situ high temperature Raman and the ^27^Al MAS NMR spectra of crystalline and as-quenched KAl(MoO_4_)_2_ were analyzed to give information on the structure transformation and Al coordination species. The results demonstrate that Al is present predominantly in [AlO_6_] octahedra in both KAl(MoO_4_)_2_ glass and melt, with the tetrahedrally coordinated Al being minor approximately at 2.7% under the fast quenched conditions in this work. The effect of K^+^, from ordered arrangement in the crystal to homogenously random distribution in the melt on the local chemical environment of Al, was also revealed. The distribution and quantitative analysis of different Al coordination subspecies are finally discussed and found to be dependent on the thermal history of the glass samples. It is of great significance for understanding the role of trivalent cations in the melt, and thus optimizing compositions and synthesis conditions of functional crystals, as well as further exploring the new possible candidates as tunable solid-state lasers for the family M^I^M^III^(M^VI^O_4_)_2_ (M^I^ = alkali metal, M^III^ = Al, In, Sc, Fe, Bi, lanthanide; M^VI^ = Mo, W).

## Figures and Tables

**Table 1:** The chemical shift values of four-, five-, and six-coordinated Al species (Al^IV^, Al^V^, and Al^VI^, respectively) in various inorganic salt systems determined by ^27^Al nuclear magnetic resonance (NMR).

Compound Family	*δ*_i_ (ppm)			References
	Al^VI^	Al^V^	Al^IV^	
(Al_0.1_Sc_0.9_)_2_(WO_4_)_3_	−9.5, −6.2, 14.6			[28]
Li_2_(Rb/Cs)_3_Al(MoO_4_)_4_			60.9~62.6	[29,30]
(La/Y)^3+^-Al_2_O_3_-B_2_O_3_-SiO_2_ glass	0	35	60	[31]
Tm^3+^: Al_2_O_3_-La_2_O_3_-SiO_2_ glass	0	30	50	[32,33,34]
amorphous La_2_O_3_-Al_2_O_3_-Ga_2_O_3_-5B_2_O_3_	−1.4~12.9	31.8~35.6	54.5~82.5	[35]
amorphous Al_2_O_3_	5~7	36~41	55	[36]
Al_2_O_3_-SiO_2_ glass	3~6	32~37	59~68	[32]
Na_2_O-Al_2_O_3_-SiO_2_ glass	19.1	30	60.4	[37]
CaO-Al_2_O_3_-SiO_2_ glass	14.7	20~25.1	57.3~63.6	[38,39]
MgO-Al_2_O_3_-SiO_2_ glass	0	~30	~50	[40,41]
(Ba/Ca)O-Al_2_O_3_-B_2_O_3_ glass	8~9	30, 39~50	50~60, 72~81	[42,43]
CaO-Al_2_O_3_-B_2_O_3_-SiO_2_ glass	0	30~35	50~60	[44,45,46]
Na_2_O-Al_2_O_3_-SiO_2_-Fe_2_O_3_-B_2_O_3_ glass			59.6	[47]
CaO-MgO-Na_2_O-Al_2_O_3_-SiO_2_ glass		~(−22 ± 1.5)	~(53 ± 1)	[48]
AgPO_3_-5Al_2_O_3_ glass	−13		41	[49]
Na_2_O-Al_2_O_3_-P_2_O_5_ glass	−10~−15	14~15	40~45	[50,51,52]
Li_1+x_Al_x_Ge_2-x_(PO_4_)_3_ glass	−14	10	40	[53,54]

**Table 2:** The atomic coordinates of KAl(MoO_4_)_2_ crystal used for calculating Raman vibrational modes by CASTEP.

Atoms	Fractional Coordinates of Atoms		
	*u*	*v*	*w*
O1	0.333333	0.666667	0.491085
O2	0.666667	0.333333	−0.491085
O3	0.148782	0.297564	0.152414
O4	−0.297564	−0.148782	0.152414
O5	0.148782	−0.148782	0.152414
O6	0.297564	0.148782	−0.152414
O7	−0.148782	−0.297564	−0.152414
O8	−0.148782	0.148782	−0.152414
Al	0.000000	0.000000	0.000000
K	0.000000	0.000000	0.500000
Mo1	0.333333	0.666667	0.232928
Mo2	0.666667	0.333333	−0.232928

**Table 3:** Experimental and calculated wavenumbers (cm^−1^) by CASTEP of the Raman vibrational modes of KAl(MoO_4_)_2_ prepared under slow cooling condition, together with proposed assignments.

Experimental	Calculated	Symmetry	Assignment	Reference [8]	
				Calculated	Assignment
1003	1002	*A*_1*g*_	*v*_s_(Al–O–Mo)	982	*v*_s_(MoO_4_^2−^) ^1^
931	909	*A*_1*g*_	*v*(Mo–O)	927	*v*_as_(MoO_4_^2−^)
801	857	*E_g_*	*v*_as_(Al–O–Mo)	802	*v*_as_(MoO_4_^2−^)
397	358	*A*_1*g*_	*δ*(Al–O–Mo)	595	*δ*_as_(MoO_4_^2−^) ^1^
375	330	*E_g_*		497	*δ*_as_(MoO_4_^2−^)
350	310	*E_g_*		336	*δ*_s_(MoO_4_^2−^)
175	166	*E_g_*	*δ*(Mo–O)	176	Translations of (MoO_4_^2−^)
	155	*A*_1*g*_	*δ*(Al–O–Mo)		

^1^ *v* and *δ* are denoted as stretching and bending vibrations, respectively. Subscripts, s and as, standard for symmetric and asymmetric vibrations, respectively.

## Supplementary Materials

The following are available online at www.mdpi.com/1996-1944/10/3/310/s1. Table S1: The calcination process for preparing crystalline KAl(MoO_4_)_2_, Table S2: The heating process used to prepare amorphous samples.
