# Supplementary material for: In-Situ Studies of Structure Transformation and Al Coordination of KAl(MoO4)2 during Heating by High Temperature Raman and 27Al NMR Spectroscopies
Source: Materials (Basel). 2017 Mar 17;10(3):310. doi: 10.3390/ma10030310 (PMC5503307; doi:10.3390/ma10030310)
Supplement: Supplementary file 1 [file materials-10-00310-s001.pdf]

Supplementary Materials: *In-Situ* Studies of Structure Transformation and Al Coordination of $KAl(MoO_4)_2$ during Heating by High Temperature Raman and ^{27}Al NMR Spectroscopies

Min Wang, Jinglin You, Alexander Sobol, Liming Lu, Jian Wang and Yingfang Xie

Table S1. The calcination process for preparing crystalline $KAl(MoO_4)_2$.

Heat Treatment	Parameter Settings of Temperature (K)	Dwell Time (min)	Crucible
1	593	120	
2	773	180	Pt
3	873	2400	
and then cooled slowly (0.24 K/min) to room temperature			

Table S2. The heating process used to prepare amorphous $KAl(MoO_4)_2$.

Heat Treatment	Parameter Settings of Temperature (K)	Dwell time (min)	Crucible
1	593	120	
2	923	120	Pt
3	1173	240	
The melt was then quenched by the "hammer-and-anvil" technique (by liquid quenching between two metallic plates).			

© 2017 by the authors. Submitted for possible open access publication under the terms and conditions of the Creative Commons Attribution (CC BY) license (http://creativecommons.org/licenses/by/4.0/).
